# Genetic Diversity of Tick-Borne Rickettsial Pathogens; Insights Gained from Distant Strains

**DOI:** 10.3390/pathogens3010057

**Published:** 2014-01-14

**Authors:** Sebastián Aguilar Pierlé, Ivan Imaz Rosshandler, Ammielle Akim Kerudin, Jacqueline Sambono, Ala Lew-Tabor, Peter Rolls, Claudia Rangel-Escareño, Kelly A. Brayton

**Affiliations:** 1 Program in Genomics, Department of Veterinary Microbiology and Pathology, Paul G. Allen School for Global Animal Health, Washington State University, Pullman, WA 99164-7040, USA; 2 National Institute of Genomic Medicine, Computational Genomics Lab, Mexico City 14610, Mexico; E-Mails: iimaz@inmegen.gob.mx (I.I.R.); crangel@inmegen.gob.mx (C.R.-E.); 3 The University of Queensland, Queensland Alliance for Agriculture & Food Innovation, St. Lucia, Queensland 4072, Australia; E-Mails: Ammielle.Kerudin@uqconnect.edu.au (A.A.K.); a.lew.tabor@uq.edu.au (A.L.-T.); 4 Queensland Department of Agriculture, Fisheries & Forestry, Tick Fever Centre, Wacol, Queensland 4076, Australia; E-Mails: jacqui.sambono@gmail.com (J.S.); Peter.Rolls@daff.qld.gov.au (P.R.)

**Keywords:** intracellular bacteria, comparative genomics, SNPs, *Rickettsiales*, *Anaplasma*

## Abstract

The ability to capture genetic variation with unprecedented resolution improves our understanding of bacterial populations and their ability to cause disease. The goal of the pathogenomics era is to define genetic diversity that results in disease. Despite the economic losses caused by vector-borne bacteria in the Order Rickettsiales, little is known about the genetic variants responsible for observed phenotypes. The tick-transmitted rickettsial pathogen *Anaplasma marginale* infects cattle in tropical and subtropical regions worldwide, including Australia. Genomic analysis of North American *A. marginale* strains reveals a closed core genome defined by high levels of Single Nucleotide Polymorphisms (SNPs). Here we report the first genome sequences and comparative analysis for Australian strains that differ in virulence and transmissibility. A list of genetic differences that segregate with phenotype was evaluated for the ability to distinguish the attenuated strain from virulent field strains. Phylogenetic analyses of the Australian strains revealed a marked evolutionary distance from all previously sequenced strains. SNP analysis showed a strikingly reduced genetic diversity between these strains, with the smallest number of SNPs detected between any two *A. marginale* strains. The low diversity between these phenotypically distinct bacteria presents a unique opportunity to identify the genetic determinants of virulence and transmission.

## 1. Introduction

Since the dawn of the genomic era in the mid-1990s, the study of pathogen biology has benefited from the wealth of information and insight provided by bacterial genomes [1]. The current age of next-generation sequencing is characterized by technological advances paired with cost reduction, which allow for investigations into genome evolution. The potential to capture whole-genome sequence variation enables us to identify the genetic alterations that characterize the adaptation of bacterial pathogens to their hosts and environments. This unprecedented resolution directly impacts our ability to understand bacterial populations and identify the genetic markers of virulence, offering new hope in the fight against infectious diseases. Whole genome sequencing (WGS) provides us with a “top-down” approach to associate genotype with phenotype and has sped up learning about the basis of virulence. New sequencing technologies have also become an essential tool in understanding the historical relationships between living organisms. Molecular phylogenetic data are instrumental in research on the history of life and on the diversity of living organisms, and genome-scale approaches are known to provide unprecedented power in precise reconstructions of historical associations [2].

*Anaplasma marginale* is a tick-transmitted rickettsial pathogen of cattle resulting in decreased production due to weight loss, abortion and lower milk yields. This obligate intracellular pathogen has a worldwide distribution, with far-reaching economic impact [3]. *A. marginale* has a small genome of 1.2 Mb for which the sequence of multiple strains has been determined, revealing a high degree of interstrain variation [4,5,6]. No strain-specific genes and no plasmids were found among *sensu stricto* strains. In contrast, a high degree of allelic diversity, characterized by a high number of single nucleotide polymorphism (SNPs), has been found in *A. marginale*. Global comparison of five strains revealed a total of 20,082 sites with SNPs identified in at least one of the analyzed strains, with an average of 6,000 SNPs found between any given pair.

Together with *Babesia bigemina* and *B. bovis*, *A. marginale* affects livestock in Australia, with more than 7 million cattle at risk. These agents are associated with $22 million in production losses in 1998, despite an $8.5 million investment in acaricides and live vaccines in Australia [7]. An alternative for anaplasmosis control in Australia emerged in the early 2000s with the characterization of an attenuated *A. marginale* strain. Identified as Dawn, this strain displayed lower or similar pathogenicity than *A. marginale subsp. centrale*, a relatively benign subspecies that is used as a live vaccine in a number of countries including Australia [8,9,10]. Steers first exposed to the Dawn strain of *A. marginale* were resistant to challenge by four field strains. Additionally, two attempts to transmit the Dawn strain with *Rhipicephalus microplus* ticks were unsuccessful. In contrast, the Gypsy Plains strain is a prototypically virulent, tick transmissible, Australian strain of *A. marginale* [11]. 

The availability of these two phenotypically distinct and geographically distant strains puts us in a unique place to analyze the genetic variation that defines the phenotypic and evolutionary diversity of *A. marginale*. Massively parallel sequencing was used to sequence the genomes of these phenotypically distinct strains to list the genetic variants associated with differences in virulence and vector transmission. This compilation of differences yielded a genetic marker that can be used to distinguish the attenuated and virulent field strains: gene *AM415*. The accessibility of additional genome sequences allowed us to study evolutionary relationships between *A. marginale* strains. Phylogenetic analyses of 11 *A. marginale* genomes revealed a marked evolutionary distance of the Australian strains from all previously sequenced strains. SNP analysis showed a strikingly reduced genetic diversity between the Australian strains, with the smallest number of SNPs ever detected between any two *A. marginale* strains (195). The small number of variants detected between the phenotypically distinct bacteria presents us with a unique opportunity to identify the genetic determinants of virulence and transmission.

## 2. Results

### 2.1. Genome Sequences

**Figure 1:**
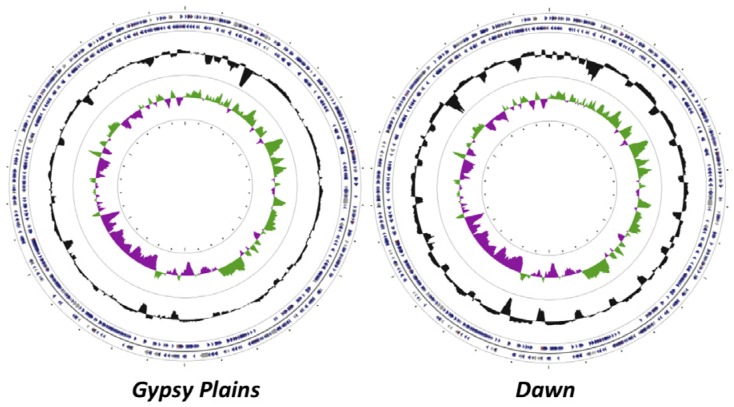
Circular display of the annotated chromosomes of the Gypsy Plains and Dawn strains of *A. marginale.* The circular representation of the Gypsy Plains and Dawn genomes are shown. CDSs are highlighted with blue colored arrows; tRNAs are highlighted with red arrows and rRNAs with grey arrows. The GC (Guanine-Cytosine) content is shown by a black histogram. The GC skew is indicated by a green histogram on the plus strand and by a purple histogram on the minus strand.

High coverage pyrosequencing data was obtained for the Gypsy Plains and Dawn strains of *A. marginale,* with 74x and 23x, respectively. These data were scaffolded against the fully sequenced St. Maries strain to yield a single contiguous pseudochromosome of 1,198,622 and 1,196,760 nucleotides for the Gypsy Plains and Dawns strains (Figure 1). There are 86 gaps in coverage in the Gypsy Plains genome and 112 gaps in the Dawn genome. Annotation of these genomes yielded a total of 959 CDSs (Coding DNA Sequences) in the Gypsy Plains strain and 901 CDSs in the Dawn strain. Fewer genes were annotated in the Dawn strain due to the lower coverage and higher number of gaps.

### 2.2. Identification of INDELs as Genetic Markers between the Attenuated and Virulent Strains

**Figure 2:**
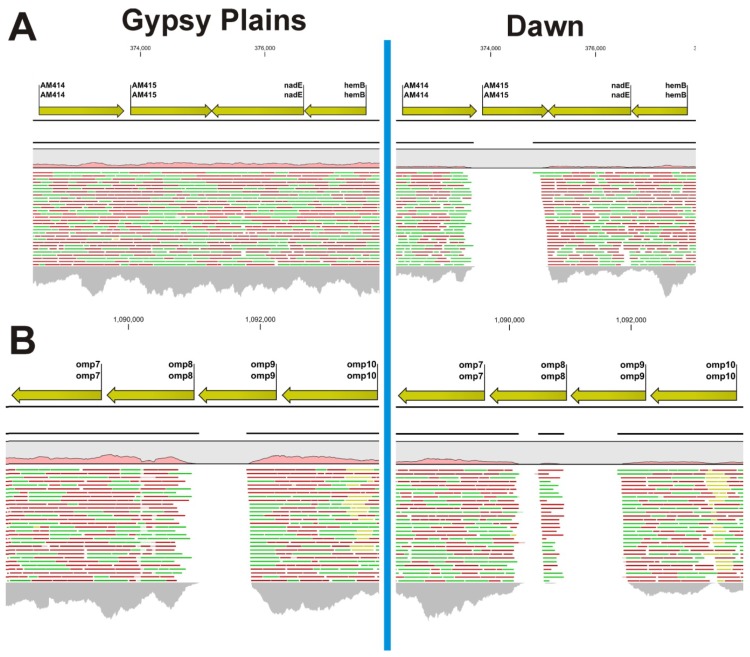
Read mapping highlights large INDELs in the Gypsy Plains and Dawn strains. Mapping of 454 reads to the St. Maries genome of *A. marginale* highlights large INDELs in the Gypsy Plains and Dawn strains. Positions in the St. Maries genome are shown on top of the figure. Genes and their annotation in the St. Maries genome are shown with yellow arrows. A pink histogram shows coverage relative to whole genome mapping. Red, green and yellow lines represent the reads mapped to that particular region of the genome. Green reads have been mapped in forward direction; red reads have been mapped in reverse direction. Yellow reads are reads that have multiple equally good alignments to the reference. The CLC4 and GxWb5.5 mapping algorithms were used with the following parameters: mismatch cost 2, insertion cost: 3, deletion cost: 3, length fraction: 50%, and similarity fraction: 80%. The grey histogram at the bottom of the figure shows coverage depth for that particular region of the genome. (**A**) Shows the INDEL found in the Dawn strain for gene *AM415*. This 1193 bp long INDEL is only found in the non-transmissible/low virulence Dawn strain. Gene *AM415* is present in the Gypsy Plains strain; (**B**) Shows the INDELs found at positions 1091010–1091800 in the Gypsy Plains strain. This INDEL affects the *omp8* gene and is 791 bp long. Two INDELs were found for the Dawn strain in the same region of the genome. These two INDELS are found at positions 1090143–1090469 and 1090890–1091782, with 327 and 892 bp in length, respectively for the Dawn strain. While the INDEL for the *AM415* gene was confirmed through PCR, amplification of the *omp6-10* operon revealed a 1.5 kb deletion common to both Australian strains. The mapping discrepancies shown in this figure are likely due to sequence diversity of the Australian *omp* genes.

Quality and probabilistic based identification of INDELs revealed a total of 150 and 159 differences in the Dawn and Gypsy Plains strains respectively when compared to the St. Maries strain as a reference. Although the vast majority of these ranged between two and eight nucleotides (Additional files 1 and 2), two longer INDELs stood out as potential candidates to differentiate these strains. The first one of these was found at positions 373659–374852 (Figure 2). This INDEL was 1194 bp long and encompassed a whole gene: *AM415*. This gene was absent in the Dawn strain but present in Gypsy Plains. Two closely positioned large INDELs were found in the Dawn strain at positions 1090143–1090469 and 1090890–1091782, with 327 and 893 bp in length, respectively (Figure 2). In the Gypsy Plains strain a single INDEL of 791 bp was found in this region at positions 1091010–1091800. These INDELs encompass parts of the Open Reading Frames (ORFs) of two genes that code for the related outer membrane proteins: *omp8* and *omp9*.

**Figure 3:**
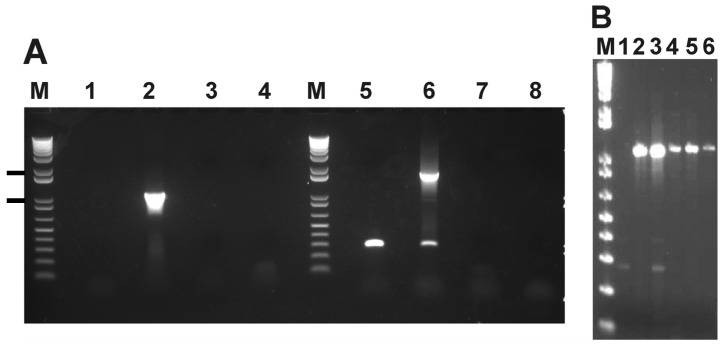
Amplification of the *AM415* INDEL in *A. marginale* strains and field isolates. Panel A shows amplification of the AM415 INDEL in the Dawn and Gypsy Plains strains with two different primer sets. Lanes 1 and 5 contain DNA from the Dawn strain, lanes 2 and 6 contain DNA from the Gypsy Plains strain, lanes 3 and 7 contain DNA from the unrelated tick-borne pathogen *Babesia bovis* and lanes 4 and 8 contain a water control (no template). Lanes marked M is the 1kb Plus DNA ladder (Life Technologies^TM^) with bands of 1,000 and 2,000 bp indicated on the left. Lanes 1–4 show results using primer set AM415 designed to sit inside of the INDEL and show the absence of amplicon in the Dawn strain. The expected size of the product was 1,027 bp in Gypsy Plains. Lanes 5–8 show results using primer set AM415L which is designed to show a size difference between the Gypsy Plains and Dawn strains by flanking the INDEL. The expected size of the products was 1,684 bp for Gypsy Plains and 331 bp for Dawn. Panel B shows usage of primer set AM415L with several virulent field isolates. Lane 1 shows the Dawn strain, lane 2 field isolate P11-72270, lane 3 shows the Gypsy Plains strain and lanes 4 through 6 show isolates P11-702600009, P11-702600001 and P11-702600005 respectively.

Two primer sets were designed for the INDEL in *AM415*: one to show the absence of this gene in the Dawn strain, and one to flank the INDEL and test it as a marker for the attenuated strain. A difference in size between virulent and attenuated strains was expected for the second primer set. Differences in size were clear for the INDEL that encompasses gene *AM415* (Figure 3). Sequencing analyses confirmed the deletion of *AM415* in the Dawn strain. The presence of a smaller amplicon in the Gypsy Plains strain, which is similar in size to the Dawn strain product, suggests that Gypsy Plains may have a heterogeneous mix of organisms. This smaller amplicon was not detected in other field strains. A deletion was detected in both Australian strains relative to North American strains when testing the INDEL located within *omp8–9* with a set of flanking primers. In North American strains, *omp8* and *9* are contained in an operon of genes encoding outer membrane proteins, in 5ʹ->3ʹ order, *omp10*, *9*, *8*, *7* and *6.*Amplification of the *omp6–10* operon revealed identically sized products for both Australian strains; these products were 1.5 kb smaller when compared to the St. Maries strain amplicon (data not shown). Although one of the *omp* genes is likely to be missing in the Australian strains, we did not further investigate this INDEL due to its failure to serve as a genetic marker and the repetitive nature of the genes involved in the *omp6–10* operon. Considering these results, the primer set designed for the *AM415* INDEL was tested using DNA from several virulent field isolates from Australia, and consistently differentiated the isolates (Figure 3).

### 2.3. SNPs that Segregate with Virulence and Transmission Phenotypes

**Figure 4:**
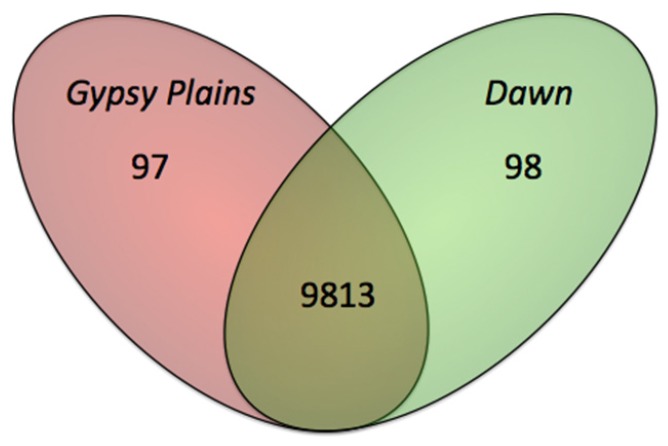
Single Nucleotide Polymorphisms (SNPs) identified between the Gypsy Plains, Dawn and St. Maries strains of *A. marginale*. The Venn diagram shows the SNPs found between the St. Maries, Gypsy Plains and Dawn strains of *A. marginale*. A total of 10,008 SNPs was identified when comparing the two Australian strains to the St. Maries strain. The sum of 9,813 SNPs were found to be common to both Australian strains when compared to St. Maries. Only 97 (shown in pink) and 98 (shown in green) of these SNPs were unique to either the Gypsy Plains or Dawn strain, respectively.

**Figure 5:**
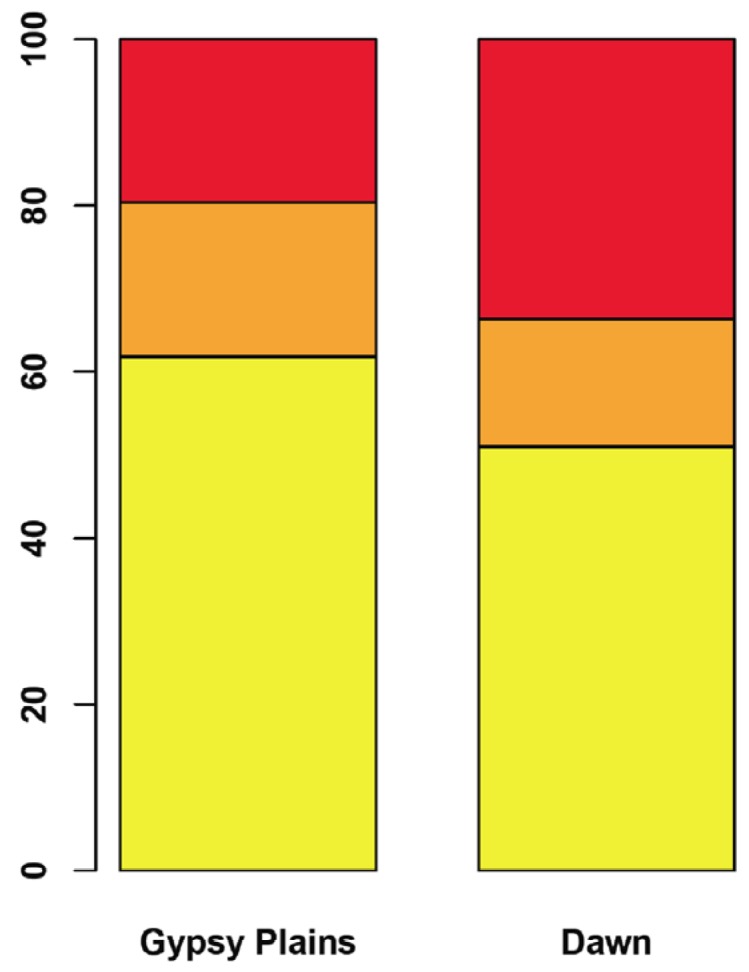
Functional classification of segregating variants The proportion of segregating variants assigned to each class as evaluated by the SNPeff algorithm in the Gypsy Plains and Dawn strains is shown. Red shows missense variants, orange shows silent variants, and variants that fell into the “none” category are shown in yellow.

Whole genome comparisons between the Dawn, Gypsy Plains and St. Maries strains identified 10,008 SNPs (Figure 4 and Additional file 3). A total of 195 of these variants were found to be unique to either the Dawn or Gypsy Plains strain. Ninety seven SNPs were unique to the Gypsy Plains strain, whereas 98 were unique to the Dawn strain. These 195 variants will be identified as segregating throughout the manuscript as they differ between the attenuated and virulent strains. The snpEff algorithm was used to assess the impact of the identified variants by assigning them into one of four categories: nonsense, missense, silent and “none” variants. The first category, nonsense variants, corresponds to point mutations that result in the creation of a new stop codon. Missense variants are variants that result in an amino acid change, also termed non-synonymous changes (NS). Silent variants are point mutations that result in a synonymous codon change, but not an amino acid change or a stop codon. The category “none” corresponds to variants that don’t fall into any of the above categories i.e. intergenic SNPs. Of the 195 segregating SNPs, 0 were classified as nonsense mutations, 52 as missense, 33 as silent and 110 as having no impact (Additional file 4). Figure 5 shows the proportion of each functional classification of variants per strain. Fisher’s exact test was used to test whether each of these categories had different proportions in the phenotypically distinct strains. The missense category was the only one to display a difference between the strains (*p* = 0.035), with a larger representation in the non-transmissible/attenuated Dawn strain.

A summary of the segregating variants identified in both strains together with their impact as evaluated by snpEff can be found in Additional file 4. The 195 segregating variants were found to be distributed in 82 different genes. Of particular interest are the variants that were unique to the Dawn strain, as variants that are exclusive to the non-transmissible attenuated strain are likely to be responsible for the phenotypes of interest. These 98 “Dawn specific” SNPs fell in 48 genes. Out of these 48 genes 37 were annotated as hypothetical proteins and only 11 had predicted functions. Of the 11 genes with predicted functions almost half encode known surface proteins: *msp1B-1*, *msp1B-2*, *virB2*, *omp11* and *msp2* [4,12]. Next, the Dawn specific SNPs were compared against a previously published list of candidate polymorphisms that segregate with transmission phenotype in seven *A. marginale* strains [13].

Fourteen of the unique Dawn SNPs segregated with the non-tick transmissible Florida strain. SNPs that segregated with the Florida strain have their position highlighted in red, SNPs that were not common to the Dawn and Florida strains are highlighted in light blue in Additional file 4. Finally, we evaluated the density of the segregating variants across the *A. marginale* chromosome, in order to see if these segregating variants were confined to specific regions or evenly distributed. Over half of the variants, 59% in the Dawn strain and 63% in the Gypsy plains strain were found to be less than 1kb apart from each other. The average distance for the variants that were less than 1kb apart was 182 and 131 bp in the Dawn and Gypsy Plains strains respectively.

### 2.4. Phylogenetic Analysis in 11 A. marginale Genomes

**Figure 6:**
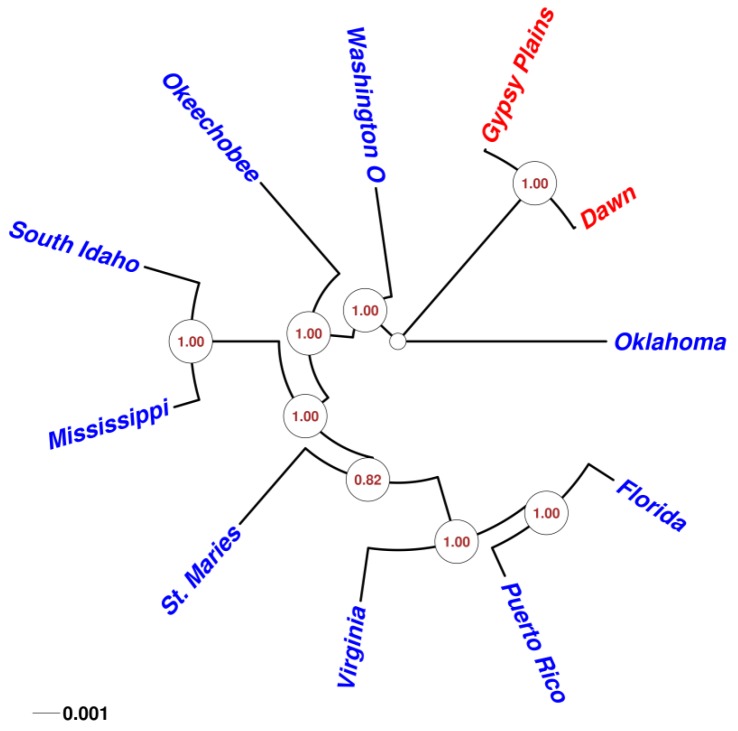
Phylogenetic analysis of 11 sequenced *A. marginale* genomes. A circular unrooted phylogenetic tree that displays the relationships between all 11 sequenced *A. marginale* genomes is shown. All but one of the clades generated were resolved in 100% of all tree calculations (1.00 alTR); this was confirmed using bootstrapping analysis. Predictably, the Australian strains, highlighted here in red, fell into their own distant branch in the tree.

Given the highly conserved nature of *A. marginale* genomes, as well as their high coding density, a phylogenetic analysis was carried through whole genome comparison of eleven different strains. A multiple alignment with 98.1% of the sites having concordance was used to build a PhyML maximum likelihood phylogenetic tree (Figure 6). All but one of the clades generated were resolved in 100% of all tree calculations (1.00 alTR), this was confirmed using bootstrapping analysis. Predictably, the Australian strains fell into their own distant branch in the tree. These strains also showed the smallest distance, consistent with the small number of variants detected through SNP analysis. Interestingly, the Oklahoma strain seems to be the closest to the Australian strains. Moreover, Oklahoma is the only *Anaplasma* strain separated from the common branch that encompasses the other American strains. 

## 3. Discussion

Since the early days of the genomic era, deductions of the variants that differentiate pathogenic microbes from their attenuated relatives were based on pairs of strains. More recently, genomic comparisons amongst multiple strains of the same species are possible and commonplace. These studies defined the concept of a pangenome for a species, reflecting the observation that the genetic content of a population exceeds that of any single individual member [14]. Among the mechanisms known to be responsible for the differences evident among bacterial species, lateral gene transfer is arguably one of the most studied. It gained notoriety in the 1950s, when multidrug resistance patterns emerged on a worldwide scale [15]. Comparative genomic analyses have revealed extensive gene transfer via insertion of phage or plasmid DNA [16]. Obligate intracellular vector-borne bacterial pathogens such as *A. marginale* are forced to generate variation on a different scale. This is due to their densely packed genomes, reduced opportunities for genetic exchange and the need to contend with protective mechanisms from at least two biologically distinct types of hosts, mammalian and arthropod [17]. Sequencing of two phenotypically distinct Australian strains of this tick transmitted pathogen highlights the idea that the genetic variation that defines a phenotypic trait can be relatively small in size and density.

Comparison of the Gypsy Plains and Dawn strains of *A. marginale* revealed the smallest number of variants between any two sequenced *Anaplasma* genomes, with 195 SNPs. This small number is intriguing considering the differences in virulence and vector transmission efficiency between these two strains of bacteria. It has been previously shown that a very small number of SNPs can drastically change a trait. Some microorganisms have been shown to shift from innocuous to pathogenic with a single base change [18,19]. The small number of differences identified between these strains that differ in their virulence and their ability to be transmitted by ticks offers opportunities for disease control and monitoring.

The small number of INDELS that were identified through comparative genomics can be exploited for diagnostic purposes. The differences in virulence reported for these Australian strains [10], and the potential of the Dawn strain as a vaccine led us to identify a molecular marker that would allow for discrimination of this relatively benign strain from virulent field strains. The *AM415*, *omp8* and *9* genes were identified as potential molecular markers between the two strains. *AM415* was confirmed as missing in the Dawn strain through PCR and sequencing. *AM415* was then confirmed as a marker by amplification in a number of field strains. Gene *AM415* is annotated as a hypothetical protein and has conserved domains for a family of sugar transporters. The deduced amino acid sequence for *AM415* has high identity (71.4%) to AM414 and is likely a duplicated gene. Because *AM414* appears to be a duplicated copy of *AM415*, the absence of *AM415* is likely not to be responsible for either of the phenotypic differences displayed by Dawn as compared to Gypsy Plains. The *omp8–9* INDEL revealed by read mapping to the St. Maries strain identified a deletion in the Australian strains as compared to the North American strains. Although the Australian version of the *omp6–10* operon is likely missing one gene, *omp8–9* differences did not constitute a good candidate for a molecular marker to discriminate the Dawn strain from virulent field isolates. These genes code for outer membrane proteins known to be expressed in both the mammalian and arthropod hosts [20]. Although operons are generally sensitive to rearrangements, recent studies have identified factors that promote their evolution including: a high population size, a high horizontal transfer rate and a low number of genes [21]. The latter seems to be the most likely contributing factor in the case of the *omp6–10* operon. The phenotypic differences seen in these Australian strains that share a collapsed *omp6–10* operon suggests redundancy of some of these surface proteins and warrants further investigation. 

The small number of single nucleotide variants detected between these strains also allows us to map genetic polymorphisms underlying phenotypic differences. When the 195 segregating variants were categorized through the snpEff algorithm a statistically significant higher proportion of missense SNPs were identified in the Dawn strain compared to the virulent/transmissible Gypsy Plains strain. These are NS variants, which is interesting in the context of the diverging phenotypes and the likelihood of a single nucleotide change having an impact. A closer inspection of the 98 variants that were unique to the non-transmissible/low virulence Dawn strain, revealed that almost half of the genes they affect with predicted functions code for known surface proteins: *msp1B-1*, *msp1B-2*, *virB2*, *omp11* and *msp2* [4,12]. These variable surface proteins have been shown to be differentially transcribed between the arthropod and mammalian hosts (manuscript submitted). Additionally, the MSP1 complex has been shown to have differential adhesion activity between vertebrate and invertebrate hosts [22]. The involvement of these surface proteins in vector transmission should be tested through targeted gene replacements [23], as their interaction with vector molecules is likely. 

Whole genome phylogenetic analysis of all sequenced *A. marginale sensu stricto* strains confirmed, as expected, that these Australian strains are the two most closely related *A. marginale* genomes available to date. One possibility is that diverse *A. marginale* strains exist in Australia but we did not capture the diversity when sequencing just two strains, however, an alternative possibility is that the relatively restricted genetic diversity is due to the fact that *A. marginale* was introduced relatively recently to Australia. *A. marginale* was likely introduced into Australia in the 1900s with the arrival of *R. microplus* ticks, and a limited introduction of the pathogen has been suggested [24]. Furthermore, although *A. marginale* can be transmitted, will grow and can be maintained in a large number of wild ruminants, such as water buffalo, white-tailed deer, mule deer and black-tailed deer [25,26], these reservoirs species were only introduced to Australia in the past 200 years [27]. This relatively recent introduction of potential reservoirs and vector species supports the genetic conservation of Australian strains. Pathogens that have been introduced to a new environment and have a shorter relationship with their hosts are, in fact, characterized by low genetic diversity. This can be due to a genetic bottleneck experienced during the process of introduction, given that only a few genotypes are usually introduced. Most bacterial models that have “recently” been introduced to a region display the expected lack of genetic diversity paired with correspondingly similar phenotypes [28].

The dissimilar phenotypes of the two sequenced strains in a highly similar genetic background is particularly intriguing. Recent studies on population genomics and bacterial lineage emergence have shown that the microorganisms that have diverged phenotypically display an unexpectedly small amount of variation [29]. This variation is mainly represented by SNPs and is independent of DNA acquisition. Furthermore, this variation was shown to be restricted to a few small regions of the core genome, instead of the expected genome wide sweeps [30]. Interestingly, the density and distribution of the segregating SNPs was also highly restricted for these Australian strains. More than half of the variants were found to be less than 1kb apart from each other in both strains. Furthermore, the average distance for these variants was 182 and 131 bp in the Dawn and Gypsy Plains strains respectively. Future studies with a larger sampling of Australian *A. marginale* are necessary to further support this idea and answer questions about population bottlenecks and expansion over time and among multiple species.

Our observations in these genetically conserved yet phenotypically distinct bacterial strains add to the body of knowledge gained on bacterial populations that seem to be differentiated with a few genomic regions that have swept through subpopulations in a milieu-specific manner and at a small polymorphic level. This differentiation driven by locus specific rather than genome wide selective sweeps is similar to that of sympatric speciation by habitat specific allelic sweeps in eukaryotes [31,32]. Despite the innate biological differences in how adaptive alleles are acquired, these results reinforce the idea that the spread in populations may follow a more uniform process in both prokaryotes and eukaryotes than previously imagined.

## 4. Experimental Section

### 4.1. Genome Sequencing/*de novo* Assembly/Annotation

Sequencing of the Dawn and Gypsy Plains strains of *A. marginale* was performed as previously described using Roche’s 454 technology [6]. Accession numbers for the Gypsy Plains and Dawn strains are: CP006846 and CP006847, respectively. Raw 454 fastq files were mapped using MIRA v3.9.16 [33] using the St. Maries genome as a backbone sequence. Once the contigs were generated, the 454 reads were mapped to the assembled contigs. Bovine sequences were then filtered from unmapped reads. *De novo* assembly was then performed using the remaining unmapped reads and contigs from the first round of mapping. These contigs were assembled into a single pseudochromosome by scaffolding against the St. Maries strain genome and inserting Xs corresponding to the number of bases present in the St. Maries strain between each of the contigs. The pseudochromosomes were post-processed with the gap5 editor [34]. CDS annotation was done using glimmer3.02 [35] and the Prokaryotic Genomes Annotation Pipeline 2.0 at NCBI [36]. Interpolated markov models [37] were trained with known genes from the *A. marginale* St. Maries strain. Finally, circular genome maps were generated with CGview server [38].

### 4.2. SNP Calling and Annotation

The Dawn and Gypsy Plains sequences were aligned against the *A. marginale* St. Maries genome using the BWA-MEM algorithm [39]. Before variant calling, pre-processing was carried out using Picard tools [40]. SNPs were called using the Genome Analysis Toolkit (GATK) [41] according to its best practices manual. In order to apply variant quality score recalibration (VQSR), a database was created using the variants with the highest quality according to established hard filters: QD <2.0, MQ <40.0 o FS >60.0, where QD is Quality by Depth: Variant confidence (from QUAL field)/unfiltered depth of non-reference samples. The QD is also normalized by event length. MQ is Mapping Quality and FS is Fisher strand bias. The snpEff algorithm [42] was used to annotate the results. Fisher’s exact two tailed test was used to test whether the proportions of functionally classified variants were different between the two strains. 

### 4.3. Identification of a Genetic Marker

Raw sequences obtained for these two strains were aligned to the *A. marginale* St. Maries strain genome using custom parameters. Whole genome alignments were scanned for regions without coverage. Quality and probability based algorithms were used for INDEL detection and confirmation [43,44]. Only INDELs with >95% frequency were taken into consideration. The reported regions were then evaluated and compared in each genome in order to identify INDELs that would distinguish the virulent from the attenuated strain. The INDELs that differed between strains were then confirmed by PCR and sequencing as previously described [13]. Primers were designed to flank the INDELs thus showing a difference in size for diagnostic purposes. The primers within the *AM415* INDEL are: AM415F: AGG TGT GGG CCA TGC TTC TGA T, and AM415R: AAG TGC ACA ACC GGA TAC ATC GCA, while those that flank it are AM415FL: GGA TGC ACG CTA GGT GGA T and AM415LR: GAC AGT CAT GAA CCT GGT GCA A. Primers RecF: CAT GGA TCA CAG ACG TTG ACG ACA GG and Omp10: GGT GTT GAA TCT CGC TGG AGA AGT GG were designed to amplify the complete *omp6-10* operon in the Australian strains. Sequencing of these regions was performed as previously described [13]. GeneMark.hmm for Prokaryotes (Version 2.8) was used to identify ORFs in the omp operons sequenced in the Australian strains using the St. Maries annotation as a model [45].

### 4.4. Phylogenetic Analysis

All complete and draft *A. marginale* genome sequences available at the NCBI website were collected, including: St. Maries: CP000030.1, Florida: CP001079.1, Viriginia: ABOR00000000.1, Puerto Rico: ABOQ00000000.1, South Idaho: AFMY00000000.1, Oklahoma: AFMX00000000.1 Okeechobee: AFMV00000000.1 and Washington O: AFMW00000000.1. Multiple alignment was performed using the Mugsy algorithm [46], including the *de novo* assembled Australian strains. A *de novo* assembly was chosen in order to not bias the phylogenetic analysis with the St. Maries strain. Poorly aligned regions were removed using the trimAL tool [47]. Robustness of phylogenetic trees was evaluated using a maximum likelihood approach. Robustness was assessed by applying an Approximate Likelihood-Ratio Test (alTR) as well as a bootstrapping technique. A GTR model for sequence evolution [48] was chosen and the tree was built using the Seaview v.4 user interface [49].

## 5. Conclusions

Sequencing and comparison of two phenotypically distinct Australian strains of *A. marginale* allowed for identification of a genetic marker: *AM415*. This gene, absent in the low virulence Dawn strain, successfully discriminates it from virulent field strains. Comparative genomics also identified a rearrangement unique to the Australian strains in the *omp6–10* operon, which codes for outer membrane proteins known to be expressed in both the mammalian and arthropod hosts. SNP analyses revealed the smallest number of variants identified between any two *A. marginale* strains. Phylogenetic analyses confirmed the genetic conservation of the Australian strains. The small amount of variants identified between these phenotypically distinct strains allowed us to list genetic polymorphisms that could be responsible for differences in virulence and transmission. The recent introduction of *A. marginale* to Australia together with the genetic conservation seen in these phenotypically distinct strains raise interesting questions with respect to the differentiation of bacterial lineages.
